# The Smallest Polyoxotungstate Retained by TRIS-Stabilization

**DOI:** 10.1021/acs.inorgchem.1c01188

**Published:** 2021-06-14

**Authors:** Nadiia I. Gumerova, Alexander Prado-Roller, Mark A. Rambaran, C. André Ohlin, Annette Rompel

**Affiliations:** † Universität Wien, Fakultät für Chemie, Institut für Biophysikalische Chemie, 1090 Wien, Austria; ‡ Universität Wien, Fakultät für Chemie, Zentrum für Röntgenstrukturanalyse und Institut für Anorganische Chemie, Zentrum für Röntgenstrukturanalyse, 1090 Wien, Austria; ¶ Umeå University, Department of Chemistry, 901 87 Umeå, Sweden

## Abstract

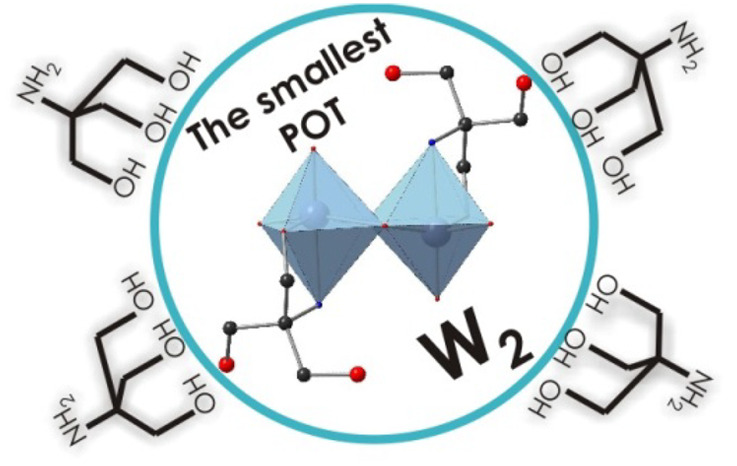

A polycondensation reaction of the orthotungstate anion WO_4_^2–^, buffered at pH 7.5 in a TRIS-HCl (0.15 M) solution, results in the first example of a discrete polyoxotungstate anion, with just two W ions stabilized with TRIS ligands. It was isolated and characterized as Na_2_[W^VI^_2_O_6_(C_4_O_3_NH_10_)_2_]·6H_2_O by single-crystal and powder X-ray diffraction, FT-IR spectroscopy, thermogravimetrical analysis (TGA), and elemental analysis in solid state and by electro-spray ionization mass spectrometry (ESI-MS), ^13^C, and ^183^W NMR, as well as Raman spectroscopy in solution. This synthesis demonstrates the crucial and new role of the added tris-alkoxy ligand in the development of a new hybrid TRIS-isopolytungstate with the lowest known nuclearity (so far) and the terminal oxygens substituted with two nitrogen atoms arising from amines of the TRIS ligands.

## Abstract 2

We report on the synthesis and characterization of a new hybrid isopolytungstate Na_2_[W^VI^_2_O_6_(C_4_O_3_NH_10_)_2_]·6H_2_O with the, so far, lowest known nuclearity and the terminal oxygen atoms substituted with nitrogen arising from amine.

Polyoxometalates (POMs) are discrete anionic molecular metal-oxide clusters that are usually composed of group V and VI transition metals in their highest oxidation states and exist at the unique interface between monomeric oxometalates and polymeric metal oxides^1^ exhibiting a wide range of applications.^2^ There are three main criteria by which a metal oxide can be called a POM: (1) addenda ions have quasi-octahedral-coordination and form d_π_-p_π_ bonds with oxygen atoms,^1^ (2) two octahedra are connected via sharing the edge, and (3) each octahedral unit has just two terminal O atoms.^3^ Oxygen atoms are the primary ligands for the addenda metals in POMs; however, replacing them with other elements while maintaining the structure is possible. So far, many synthesis procedures of oxo-replaced POM structures require water-free organic solvents and the overwhelming majority of POMs with nitrogen atoms bound to the addenda ion are polyoxomolybdates (POMos)^4^ or polyoxovanadates (POVs).^5^ Attempts to directly functionalize [W_6_O_19_]^2–^ polyoxotungstate (POT) have failed when applying imido approaches suitable for POMos and POVs, since [W_6_O_19_]^2–^ does not react with phosphinimines, isocyanates, or primary amines.^4^

In solution, small and stable POMs are interesting as useful building blocks for constructing huge metal–oxo clusters.^6^ In many cases, polycondensation reactions of [MO_*x*_]^n-^ (M = addenda ion) immediately lead to larger structures, and POMs with low nuclearity are quite elusive.^1^ In acidified solutions of WO_4_^2–^, both in the presence or absence of heteroions, anions with less than six W are underrepresented.^7−10^ The smallest discrete isopolytungstates (IPOT) verified to exist both in the solid state and in solutions are the Lindqvist hexatungstate [W_6_O_19_]^2–^ in nonaqueous media and heptatungstate [W_7_O_24_]^6–^ in water.^1,7^ Discrete binuclear complexes of pentavalent tungsten [W^V^_2_O_4_(Y)]^2−^ (Y^4−^ = hexadentate ligand, e.g., ethylenediaminetetraacetate) in octahedral coordination are known;^11^ however, they have not been obtained by acidification of orthotungstate. The strategy of using organic ligands to inhibit the formation of large clusters has previously been applied to POMos^12^ and POVs^13^ while it has not been reported for POTs. Tris(hydroxymethyl)aminomethane ((HOCH_2_)_3_CNH_2_, TRIS) has been recently utilized to stabilize and isolate elusive heptavanadate.^13^ While in biochemistry, TRIS (p*K*_a_ 8.06) is used as pH buffer between 7.0 and 9.2,^14^ in POM chemistry, TRIS is usually used for covalent organic functionalization via alkoxo-groups −CH_2_OH attachment to the POM,^15−17^ and −NH_2_ plays the key role for postfunctionalization through amide bond formation.^18−20^ Recently, we expanded the role of TRIS by showing that TRIS, as part of a buffer solution that is normally considered unimportant, plays a defining role in the formation of a new member of the Keggin family.^21^ In POM chemistry, TRIS has never acted as a primary amine by replacing oxo-ligands and thus as a protective ligand to prevent the formation of POTs with a higher nuclearity.

A discrete small anion, which is additionally organically functionalized for better stability, could be an ideal candidate for its use as a building block. To synthesize such an anion, we investigated an IPOT formation in a TRIS buffered solution (pH 7.5, 0.15 M) of WO_4_^2–^. Herein, we report for the first time the successful synthesis of the discrete [W^VI^_2_O_6_(C_4_O_3_NH_10_)_2_]^2–^ (**W**_**2**_), which meets all the requirements to be called a POM^1^ (W^VI^ has quasi-octahedral-coordination; two octahedra are connected via sharing the edge; W^VI^ has just two terminal O atoms) and is the smallest POT hybridized with TRIS known so far. **W**_**2**_ is a representative of a POM family with replaced oxygen ions by other nonmetals and the first POT functionalized directly by a primary amine in aqueous solution.

Initially, 12 mL of an aqueous solution of WO_4_^2–^ (0.188 M) was acidified with HCl (1 M) to pH 4.4 followed by the addition of TRIS (0.3 g, 2.5 mmol, 0.15 M) that led to an increase in pH to 7.5 (Figure 1A). After the final mixture was heated for 1 h at 90 °C and kept at room temperature, colorless crystals of Na_2_[W^VI^_2_O_6_(C_4_O_3_NH_10_)_2_]·6H_2_O (**Na**_**2**_**W**_**2**_) were formed. Single crystal structure analysis of **W**_**2**_ revealed that TRIS acts not only as a buffer component but also as a shielding ligand, preventing formation of IPOTs with a higher nuclearity in unbuffered WO_4_^2–^ solution, namely, H_*x*_[W^VI^_12_O_40_(OH)_2_]^(10-*x*)–^ (*x* = 0–3) and [H_2_W^VI^_12_O_40_]^6–^ between pH 2 and 5 as well as [W^VI^_7_O_24_]^6–^ and [W^VI^_12_O_40_(OH)_2_]^10–^ at pH 7.5 (Figure 1B).^7−9^

**Figure 1:**
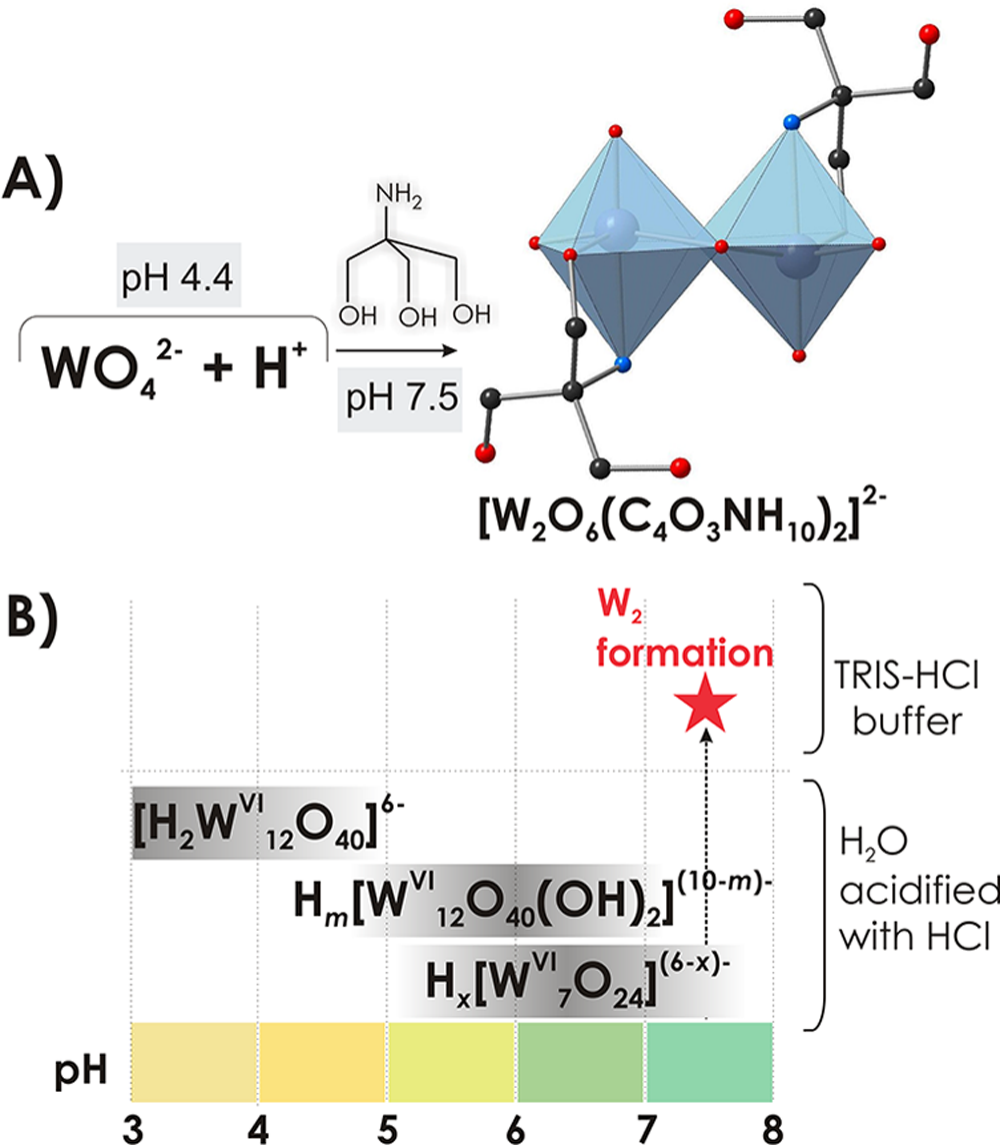
Synthetic route to prepare **W_2_** (**A**) and the speciation diagram in buffered and unbuffered^7^ aqueous solution of WO_4_^2−^ in the pH range from 3 to 8 (**B**). Color code: {WO_6_}, light blue; O, red; N, blue; C, black.

**Na**_**2**_**W**_**2**_ crystallizes in the triclinic space group *P*1̅ (CCDC 2078090). The structure is composed of a [W^VI^_2_O_6_(C_4_O_3_NH_10_)_2_]^2–^ and two Na^+^, connected to the tungsten cluster via O–CH_2_ groups of TRIS and forming a {NaO_5_} polyhedron (Figure 2). The coordination environment of each W^VI^ consists of two terminal O_t_ (*d*(W1–O4) = 1.766 Å, *d*(W1–O5) = 1.767 Å), two bridging μ_2_-O (*d*(W1–O6) = 1.858 and 2.167 Å), one oxygen atom, and one nitrogen atom (*d*(W1–N1) = 2.344 Å) from one TRIS molecule (Figure 2). The W–N bond lengths in **Na**_**2**_**W**_**2**_ are slightly longer than those previously reported in POTs (2.13 to 2.17 Å),^22,23^ indicating the weaker π contribution of the bonds.^24^ The W–W bond length is 3.194 Å, which is shorter than that in classical IPOTs.^9,21^

**Figure 2:**
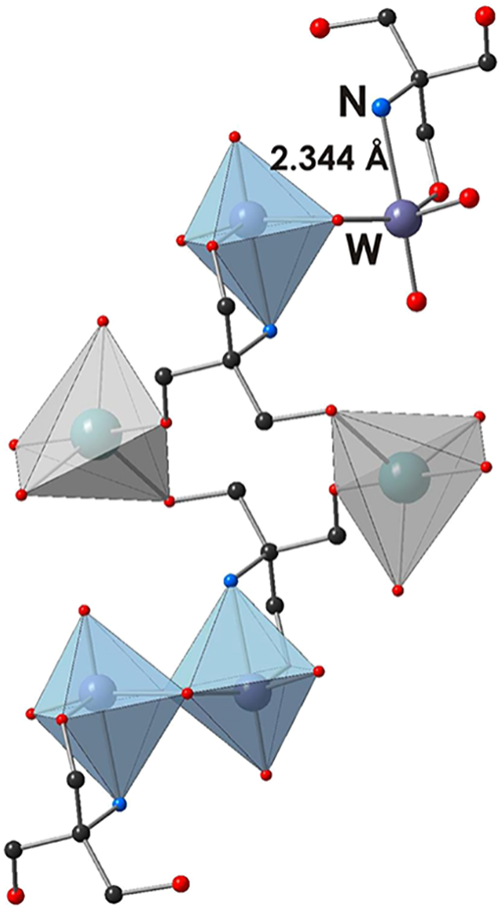
Binding of **W**_**2**_ to Na^+^ through the −CH_2_OH groups of TRIS ligands. Color code: W, purple; {WO_6_}, blue; {NaO_5_}, gray; O, red; N, blue; C, black.

The stretching vibrations of the W=O units are present at 919 cm^–1^ in the IR spectrum of Na_2_**W**_**2**_ (Figure S1). The bands at 894 cm^–1^ and in the region from 470 to 750 cm^–1^ correspond to the antisymmetric and symmetric deformation vibrations of W–O–W. The three bands at 1084, 1059, and 1040 cm^–1^ are assigned to C–O stretching vibrations, indicating the successful grafting of TRIS. TGA was used to examine the weight loss and thermal stability of the synthesized hybrid POT. The TG curve shows four weight-loss regions up to 700 °C due to dehydration followed by disintegration of TRIS (Figure S2). The experimental and simulated X-ray diffraction patterns of Na_2_**W**_**2**_ (Figure S3) fit perfectly in the range from 10 to 50° 2θ confirming its homogeneity.

The stability of **W**_**2**_ in H_2_O at pH 6.8 was investigated by ESI-MS (Figures 3, S4, S5, and Table S2). The ESI-MS spectrum recorded in positive mode exhibits three series of peaks’ envelopes at *m*/*z* between 400 and 850, which can be unambiguously assigned to the singly charged cations (Figure 3, Table S2). The first group of signals (*m*/*z* = 439.1, 461.1, 479.1, 501.0) corresponds to the monomeric complex [W^VI^(C_4_O_3_NH_8_)_2_]^0^ in which two TRIS are coordinated to W^VI^ via −CH_2_O- groups (blue in Figure 3). The signals at 711.0, 733.0, and 755.0 *m*/*z* correspond to the **W**_**2**_ (purple in Figure 3) in which one more –CH_2_OH fragment is attached to one of the equivalent W^VI^. The **W**_**2**_ with the symmetrical attachment of two additional –CH_2_OH (green in Figure 3) gives signals at 792.1, 814.1, 832.1, and 854.1 *m*/*z*. The attachment of TRIS ligands through three functional groups to the trimeric face of W^VI^ ions is a known strategy in POM chemistry for elusive anion stabilization^13^ and may be the reason that these compounds are detected in solution. The ESI-MS spectrum of Na_2_**W**_**2**_ recorded in the negative mode (Figure S4) demonstrates the absence of signals from any IPOTs.

**Figure 3:**
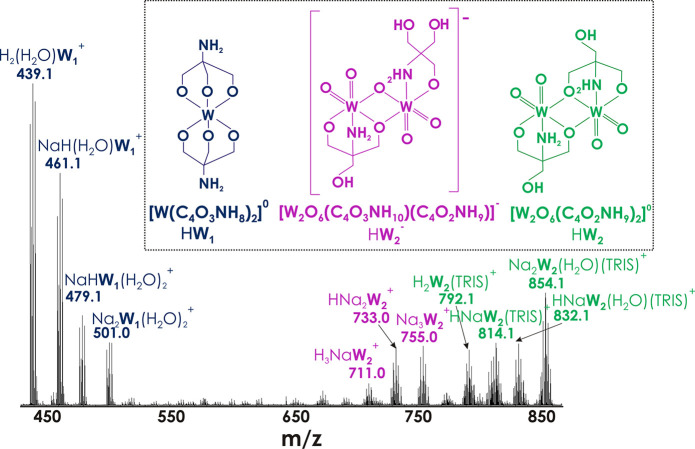
ESI mass spectrum of Na_2_**W**_**2**_ in H_2_O (pH 6.8) recorded in the positive mode in the range from 430 to 860 *m*/*z*. Three types of species are shown in blue, purple, and green. Figure S5 shows the spectrum in the range from 100 to 1000 *m*/*z*, and Table S2 provides all species with experimental and theoretical *m*/*z* values.

To further examine the solution behavior of **W**_**2**_, ^13^C and ^183^W NMR spectroscopic studies were performed in D_2_O at pH 7.5 (Figures S6 and S7). The ^183^W NMR spectrum of Na_2_**W**_**2**_ (0.058 M) in D_2_O shows one intense signal with a chemical shift at −3.4 ppm and one minor signal at −91.9 ppm (Figure S6A). Since there exist no reference spectra for POTs with two equivalent W atoms and the chemical shift in **Na**_**2**_**W**_**2**_ spectrum is very close to 0 ppm (reference Na_2_WO_4_), a ^183^W NMR spectrum for the equimolar mixture of **W**_**2**_ and WO_4_^2–^ was acquired. The spectrum of the mixture demonstrates the same two signals at −2.8 and −91.6 ppm and one additional signal at −53.4 ppm (Figure S6B). The signal at −91.6 ppm can be attributed to [W^VI^_7_O_24_]^6–^, which gives three signals at 268.8, −90.9, and −180.2 (Figure S6D). The spectrum of **W**_**2**_ recorded in NaOAc/50% D_2_O at pH 6 (Figure S6C), where no signal for WO_4_^2–^ should be observed (Figure 1B), shows a very intense signal at −2.1 ppm. DFT calculations have been performed to assign the peaks to tungsten species (Table S3). A comparison of calculated and experimental values for [W^VI^_6_O_19_]^2–^, WX_6_ (X = F^–^, Cl^–^, CO) and for **W**_**2**_ shows that the calculated shift of −17 ppm can be attributed to the signal around 0 ppm if assigned to **W**_**2**_. The ^13^C NMR spectrum of Na_2_**W**_**2**_ in D_2_O (pH 7.5) shows two signals at 59.8 and 60.8 ppm (Figure S7B), which do not correspond to the **W**_**2**_ structure observed in the solid state, where three types of carbons are present (Figure 2). However, the signals at 59.8 and 60.8 ppm also cannot be attributed to the free TRIS with signals at 56.4 and 63.0 ppm (Figure S7A). In the Na_2_**W**_**2**_ solution at pH 6, the ^13^C NMR spectrum shows three signals at 59.3, 61.4, and 63.4 ppm, which indicate potential ligand binding according to the **W**_**2**_ structure (Figure 2). Raman spectroscopy was performed in the solid state and in solution to support ESI-MS and NMR spectroscopic data. The Raman spectrum of Na_2_**W**_**2**_ in H_2_O (pH 7.5) points to its dissociation to orthotungstate WO_4_^2–^ (Figure 4). On the basis of DFT calculations, ESI-MS, and NMR and Raman spectroscopy, we argue that **W**_**2**_ is unstable in an aqueous solution at pH 7.5; however, some intermediates detected by ESI-MS (Figure 3) with another type of TRIS attachment are at least partially present in slightly acidic solutions.

**Figure 4:**
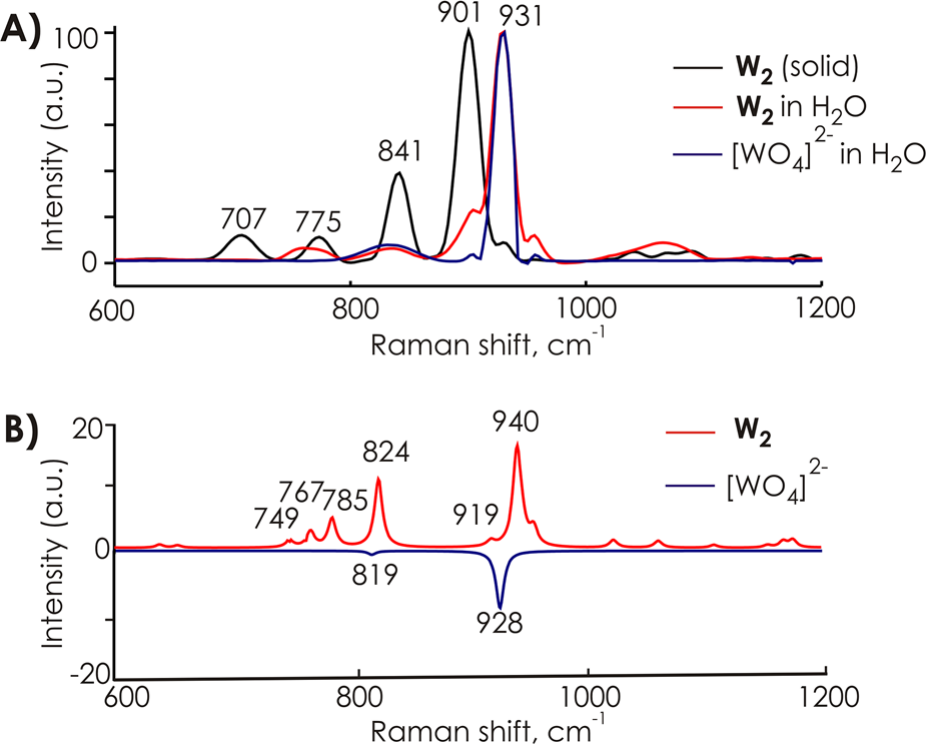
Experimental (**A**) and computed (**B**) Raman spectra of Na_2_[W^VI^_2_O_6_(C_4_O_3_NH_10_)_2_]·6H_2_O (**Na**_**2**_**W**_**2**_) and Na_2_WO_4_ in the range from 600 to 1200 cm^–1^. Solution spectra were recorded in H_2_O with pH 7.5 in **Na**_**2**_**W**_**2**_.

Considering the facile and reproducible synthesis of **W**_**2**_, as well as its small size, there are two scenarios for the use of **W**_**2**_ in the future for the formation of novel metal-oxide-based materials. One possible route is based on the rigid **W**_**2**_ nature, which is similar to that of the dinuclear [M^V^_2_S_2_O_2_(H_2_O)_6_]^2+^ (M = Mo or W),^25,26^ rendering **W**_**2**_ a potential connecting building block. The second scenario aims to expand the existing oxo-replaced POM chemistry. The strategy using the reaction of lacunary Keggin anion [PW_11_O_39_]^7–^ with the mononuclear imido-tungsten precursor [W^VI^(NC_6_H_5_)Cl_4_] was successfully applied^27^ and can be taken as a model for **W**_**2**_.

In conclusion, the existence of the anion just with two tungsten ions, which fulfills all criteria to be called POM, has been demonstrated for the first time, where W(VI) ion is coordinated to two TRIS molecules through W–N and W–O chemical bonds. Full characterizations in the solid state and in solution elucidate the composition and solution behavior of the **W**_**2**_ anion.

## Supporting Information Available

Synthetic details and spectroscopic data along with structural characterizations of the new compound (PDF)

## Accession Codes

CCDC 2078090 contains the supplementary crystallographic data for this paper. These data can be obtained free of charge via www.ccdc.cam.ac.uk/data_request/cif, or by emailing data_request@ccdc.cam.ac.uk, or by contacting The Cambridge Crystallographic Data Centre, 12 Union Road, Cambridge CB2 1EZ, UK; fax: + 44 1223 336033.

## Dedication

Dedicated to Dr. sc. nat. Hans-Joachim Lunk on the occasion of his 80th birthday.
