# Supplementary material for: The Smallest Polyoxotungstate Retained by TRIS-Stabilization
Source: Inorg Chem. 2021 Jun 14;60(17):12671–5. doi: 10.1021/acs.inorgchem.1c01188 (PMC8424642; doi:10.1021/acs.inorgchem.1c01188)
Supplement: Supplementary file 1 — ic1c01188_si_001.pdf [file ic1c01188_si_001.pdf]

Supplementary Information

The smallest Polyoxotungstate retained by TRIS-stabilization

Nadiia I. Gumerova,[†] *Alexander Prado-Roller,*[‡] *Mark A. Rambaran,*[¶] *C. André Ohlin,*[¶] *Annette Rompel**[†]

[†] Universität Wien, Fakultät für Chemie, Institut für Biophysikalische Chemie, 1090 Wien, Austria. www.bpc.univie.ac.at

[‡] Universität Wien, Fakultät für Chemie, Zentrum für Röntgenstrukturanalyse und Institut für Anorganische Chemie, 1090 Wien, Austria

[¶] Umeå University, Department of Chemistry, 901 87 Umeå, Sweden. www.moleculargeo.chem.umu.se

1. Materials and methods **2**

2. X-ray Crystallography **3**

3. IR spectrum of $Na_2[W_2O_6(C_4O_3NH_{10})_2]\cdot 6H_2O$ (Na_2W_2) **5**

4. Thermogravimetric analysis of $Na_2[W_2O_6(C_4O_3NH_{10})_2]\cdot 6H_2O$ (Na_2W_2) **6**

5. Powder X-ray diffraction **7**

6. ESI-MS **7**

7. NMR spectroscopy **9**

1. Materials and methods

The chemicals were reagent grade and used as purchased from Merck (Austria) and VWR (Austria) without further purification.

Physical methods

The *infrared spectrum* (4000−400 cm^{-1}) of $Na_2[W_2O_6(C_4O_3NH_{10})_2]\cdot 6H_2O$ (**Na_2W_2**) was recorded on a Bruker Vertex 70 IR Spectrometer equipped with a single-reflection diamond-ATR unit. *Elemental microanalysis* of C/H/N/O contents was performed by Mikroanalytisches Laboratorium (University Vienna, Faculty of Chemistry). An elemental analyzer 3000 (Eurovector) was used for C/H/N/S-analysis. O-determination was performed by high temperature digestion using the HT 1500 (Hekatech, Germany) pyrolysis system in combination with the EA 3000 system. Thermal characteristics were determined by thermal gravimetric analysis (TGA) with a Mettler SDTA851e Thermogravimetric Analyzer under air flow with a heating rate of 5 K min^{-1} in the region 298−1023 K. *Mass spectrometry* was performed with an ESI−Qq−oaRTOF supplied by Bruker Daltonics Ltd. Bruker Daltonics Data Analysis software was used for peaks assignment. The measurement was carried out in H_2O, collected in negative and positive ion mode and with the spectrometer calibrated with the standard tune−mix to give an accuracy of ca. 5 ppm in the region of m/z 300−1900. *X-ray powder diffraction* was performed in a range $10^o \leq 2\theta \leq 50^o$ on a Bruker D8 advance diffractometer equipped with Cu K*α* radiation, λ = 1.54056 , Lynxeye silicon strip detector and SolX energy dispersive detector, variable slit aperture with 12 mm. *^{183}W NMR spectra* were recorded with a Bruker FT-NMR spectrometer Avance Neo 500 MHz in 10 mm tubes for a total experiment time of ca. 60 h, using standard pulse programs at 20.836 MHz and a 63° flip angle with 1 s of relaxation delay; the temperature was kept at 25 °C. Chemical shifts were measured relative to external aqueous 1 M Na_2WO_4. *^{13}C NMR spectra* were recorded with a Bruker AV-600 spectrometer at 600.25 MHz and 25 °C Chemical shifts were measured relative to external tetramethylsilane (TMS). *Raman spectra* in the solid state were acquired on a Cora 5600 Raman spectrometer with a 532 nm laser at 50 mW. Solution phase Raman spectra were collected on 0.2 g of $Na_2[W_2O_6(C_4O_3NH_{10})_2]\cdot 6H_2O$ or Na_2WO_4 in 0.5 ml deionized water that was degassed in N_2 for 30 minutes. For the Raman calculations Gaussian 16 rev. A.03[1] using the hybrid exchange–correlation functional functional[2] and the all-electron TZP basis set by Jorge *et al.*,[3] on structures optimized at the PBE0/def2-tzvp level of theory.[4]

Synthesis of $Na_2[W_2O_6(C_4O_3NH_{10})_2]\cdot 6H_2O$ (**Na_2W_2**).

A sample of $Na_2WO_4\cdot 2H_2O$ (0.99 g, 3 mmol) was dissolved in water (12 mL) and HCl [1.0 M] (4 mL) was added, resulting in a solution with pH 4.4. The addition of tris(hydroxymethyl)aminomethane TRIS (0.3 g, 2.4 mmol) increased pH to 7.5. The reaction mixture was heated to 90 °C and stirred for 1 h, cooled to room temperature and left covered

with parafilm. Colorless block crystals were isolated after 2 weeks. Yield: 210 mg (16 % based on W). Elemental analysis found (calculated) for $Na_2W_2O_{18}C_8N_2H_{32}$: C 10.98 (11.20), H 3.78 (3.76), N 3.14 (3.26), O 33.17 (33.57). Elements ratio found (calculated): C : H : N : O = 4.08 (4) : 16.70 (16) : 1 (1) : 9.25 (9). IR (Figure S1): 3324 (s), 3251 (s), 3199 (b, s), 2941 (s), 2921 (s), 2908 (s), 2877 (s), 1668 (w), 1637 (w), 1573 (m), 1485 (w), 1463 (w), 1450 (w), 1439 (w), 1398 (w), 1382 (m), 1311 (m), 1276 (m), 1220 (m), 1184 (m), 1085 (s), 1056 (s), 1041 (s), 921 (m, sh), 892 (s), 839 (s), 777 (s), 752 (s), 710 (s), 681 (s), 623 (s), 611 (s), 598 (s), 577 (s), 501 (s), 488 (m, sh), 467 (s), 423 (s), 407 (s) cm^{-1}. The maximum solubility of $Na_2[W_2O_6(C_4O_3NH_{10})_2]\cdot 6H_2O$ (**Na_2W_2**) in H_2O and 0.1 M TRIS (pH 7.5) buffer is 50 mg/mL.

To explore the conditions of **Na_2W_2** formation, we varied the pH of the initial solution from 1.5 to 7.8 and reduced the amount of TRIS to 0.2 g (1.67 mmol, 0.1 M), while the final pH of the solution after adding the ligand was in the range between 6.6 and 8.6. Under all conditions **Na_2W_2** was formed, which confirms the stability of this small POT in the presence of TRIS.

2. X-ray Crystallography

X-ray data were collected on a Bruker D8 Venture equipped with a multilayer monochromator, MoKα INCOATEC micro focus sealed tube and Kryoflex cooling device. The structure was solved by direct methods and refined by full-matrix least-squares. Non-hydrogen atoms were refined with anisotropic displacement parameters. Hydrogen atoms were inserted at calculated positions and refined with riding coordinates. The following software was used for the structure solving procedure: Frame integration, Bruker SAINT software package[5] using a narrow-frame algorithm (absorption correction), SADABS[6] (structure solution), SHELXS-2013[7] (refinement), SHELXL-2013,[7] OLEX2,[8] SHELXLE[9] (molecular diagrams). Experimental data and CCDC-codes are listed in Table **S1**.

Table S1. Crystallographic data of $Na_2[W_2O_6(C_4O_3NH_{10})_2]\cdot 6H_2O$ (**Na_2W_2**).

Empirical formula	$C_8H_{32}N_2Na_2O_{18}W_2$
CCDC-Code	**2078090**
Formula weight M_r	858.03
Crystal system	triclinic
Space group	$P\bar{1}$
T, K	100
a, b, c (Å)	7.1695(5), 9.0714(6), 10.0065(7)
α, β, γ (°)	64.517(2), 71.339(2), 89.512(2)
V (Å^3)	550.22(7)

Z	1
D_{calc}, g/cm^3	2.590
μ, mm^{-1}	10.572
Abs. correction type	multi-scan
Abs. correction T_{min}	0.5177
Abs. correction T_{max}	0.3882
F(000)	408
Crystal size, mm	0.05× 0.05 × 0.05
Theta range for data collection	2.52 – 62.53
Index ranges	$-11 \le h \le 11$<br>$-14 \le k \le 14$<br>$-16 \le l \le 16$
Reflections collected	24516
Independent reflections	5075
R_{int}	0.0399
Data / restraints / parameters	5075/0/172
Goodness-of-fit on F^2	1.083
Final *R* indices	R_F = 0.0185, wR^2 = 0.0389 (all data)<br>R_F = 0.0170, wR^2 = 0.0380 (I>2σ(I))
Largest diff. peak and hole, e. Å^{-3}	0.180/-1.734

3. IR spectrum of $Na_2[W_2O_6(C_4O_3NH_{10})_2]·6H_2O$ (Na_2W_2)

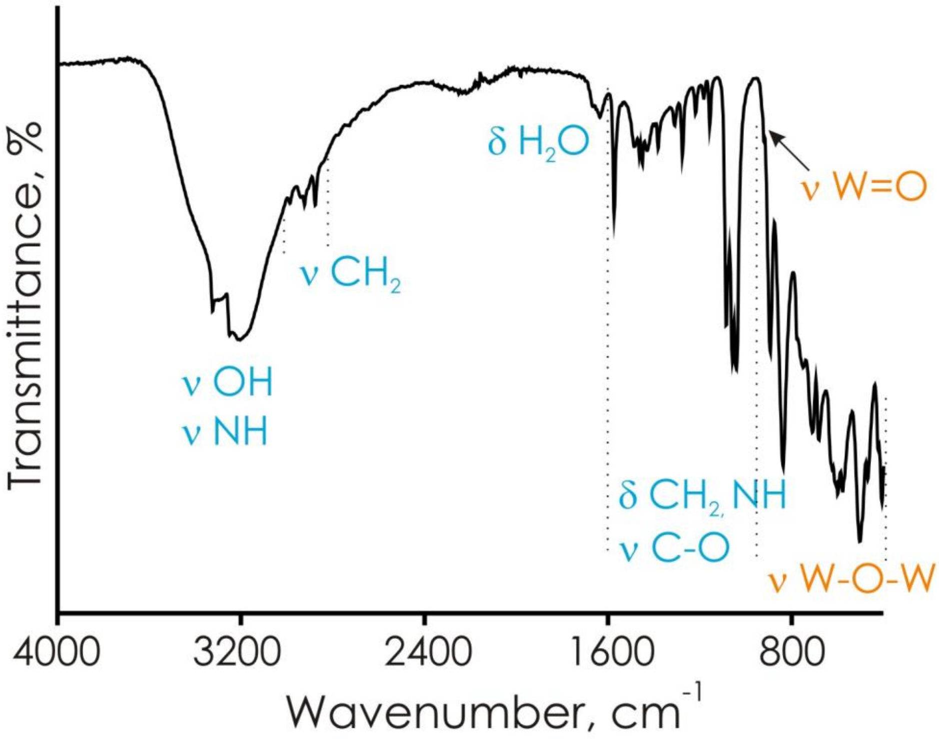

Figure S1. IR spectrum of $Na_2[W_2O_6(C_4O_3NH_{10})_2]·6H_2O$ (**Na_2W_2**) in the range from 4000 to 400 cm^{-1}.

4. Thermogravimetric analysis of $Na_2[W_2O_6(C_4O_3NH_{10})_2]·6H_2O$ (Na_2W_2)

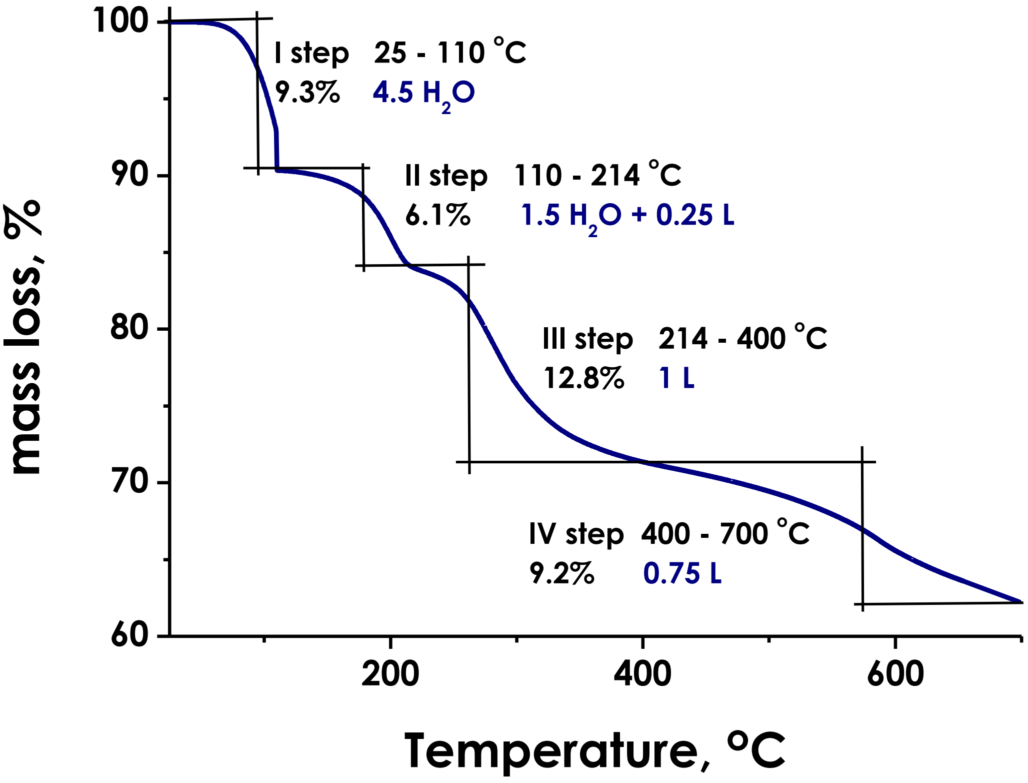

Figure S2. Thermogravimetric curve of $Na_2[W_2O_6(C_4O_3NH_{10})_2]·6H_2O$ **Na_2W_2** in the temperature region 25 – 700 °C with a heating rate of 5 °C min^{-1}. Isothermal equilibration was performed at 110 °C for 10 minutes. L stands for attached TRIS ligand without one H in the structure – $(C(CH_2OH)_2(CH_2O)NH_2)$.

5. Powder X-ray diffraction

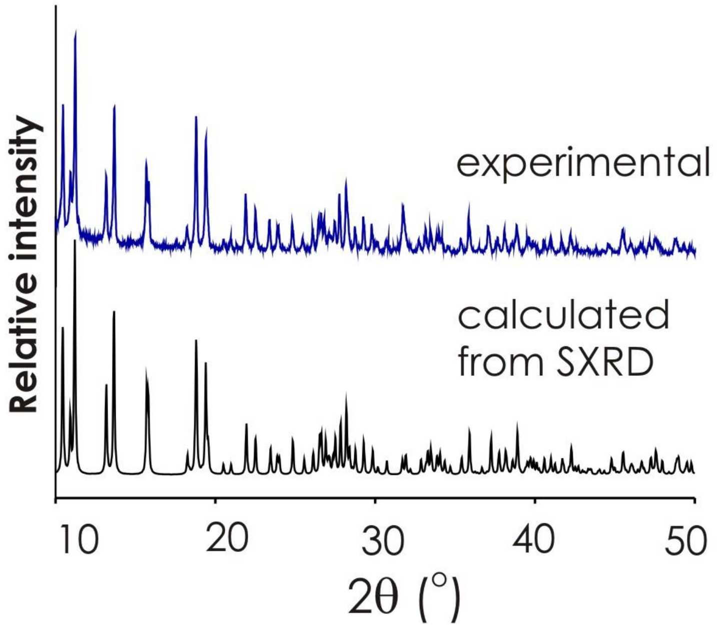

Figure S3. Experimental (blue) and simulated (black) X-ray diffraction patterns of $Na_2[W_2O_6(C_4O_3NH_{10})_2]\cdot6H_2O$ (**Na_2W_2**).

6. ESI-MS

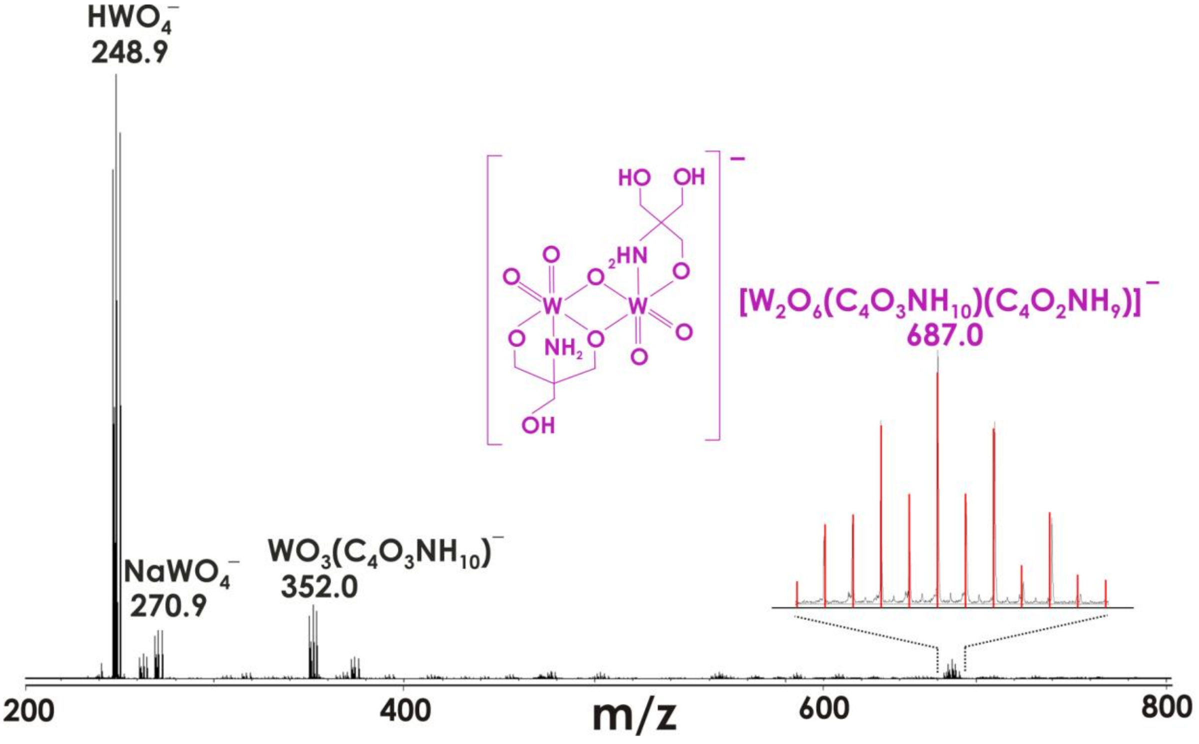

Figure S4. ESI mass spectrum of $Na_2[W_2O_6(C_4O_3NH_{10})_2]\cdot6H_2O$ (**Na_2W_2**) in H_2O (pH 6.8) recorded in a negative mode. The envelope of $[W_2O_6(C_4O_3NH_{10})(C_4O_3NH_{10})]^-$ at 687.0 m/z is shown in black with the corresponding simulated spectra shown in red.

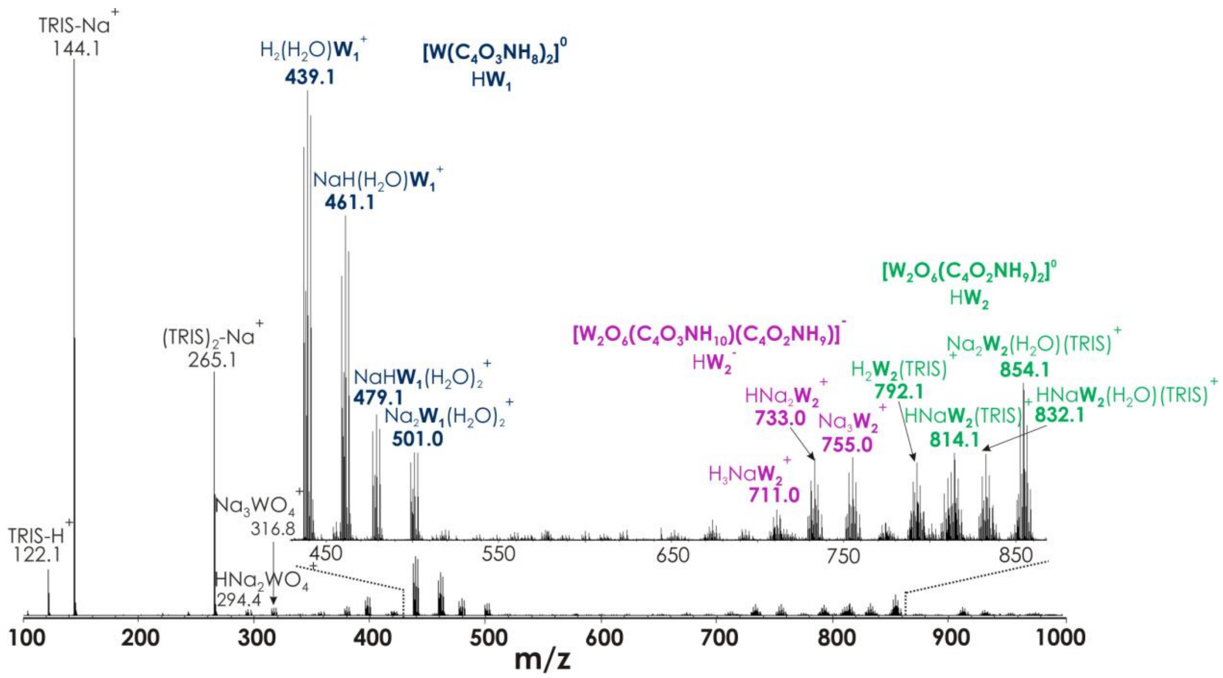

Figure S5. ESI mass spectrum of Na_2**W_2** in H_2O (pH 6.8) recorded in a positive mode in the range from 100 to 1000 m/z.

Table S2. Peak assignment in the ESI-MS spectrum $Na_2[W_2O_6(C_4O_3NH_{10})_2]\cdot 6H_2O$ (**Na_2W_2**) in H_2O (pH 6.8) recorded in positive mode (Figure S5).

Cation	Calculated m/z	Experimental m/z
$H(C_4O_3NH_{11})^+$ (TRIS-H^+)	122.1	122.1
$Na(C_4O_3NH_{11})^+$ (TRIS-Na^+)	144.1	144.1
$Na(C_4O_3NH_{11})_2^+$ ($(TRIS)_2$-Na^+)	265.1	265.1
$HNa_2WO_4^+$	294.4	294.4
$Na_3WO_4^+$	316.8	316.8
$H[W(C_4O_3NH_8)_2]^+$	421.1	421.1
$H[W(C_4O_3NH_8)_2](H_2O)^+$	439.1	439.1
$Na[W(C_4O_3NH_8)_2](H_2O)^+$	461.1	461.1
$Na[W(C_4O_3NH_8)_2](H_2O)_2^+$	479.1	479.1
$Na_2[W(C_4O_3NH_7)(C_4O_3NH_8)](H_2O)_2^+$	501.0	501.0
$HNa[W_2O_6(C_4O_3NH_{10})(C_4O_2NH_9)]^+$	711.0	711.0
$Na_2[W_2O_6(C_4O_3NH_{10})(C_4O_2NH_9)]^+$	733.0	733.0
$Na_3[W_2O_6(C_4O_3NH_9)(C_4O_2NH_9)]^+$	755.0	755.0
$H[W_2O_4(C_4O_3NH_9)_2](TRIS)^+$	792.1	792.1
$Na[W_2O_4(C_4O_3NH_9)_2](TRIS)^+$	814.1	814.1
$Na[W_2O_4(C_4O_3NH_9)_2](TRIS)(H_2O)^+$	832.1	832.1
$Na_2[W_2O_4(C_4O_3NH_9)(C_4O_3NH_8)](TRIS)(H_2O)^+$	854.1	854.1

7. NMR spectroscopy

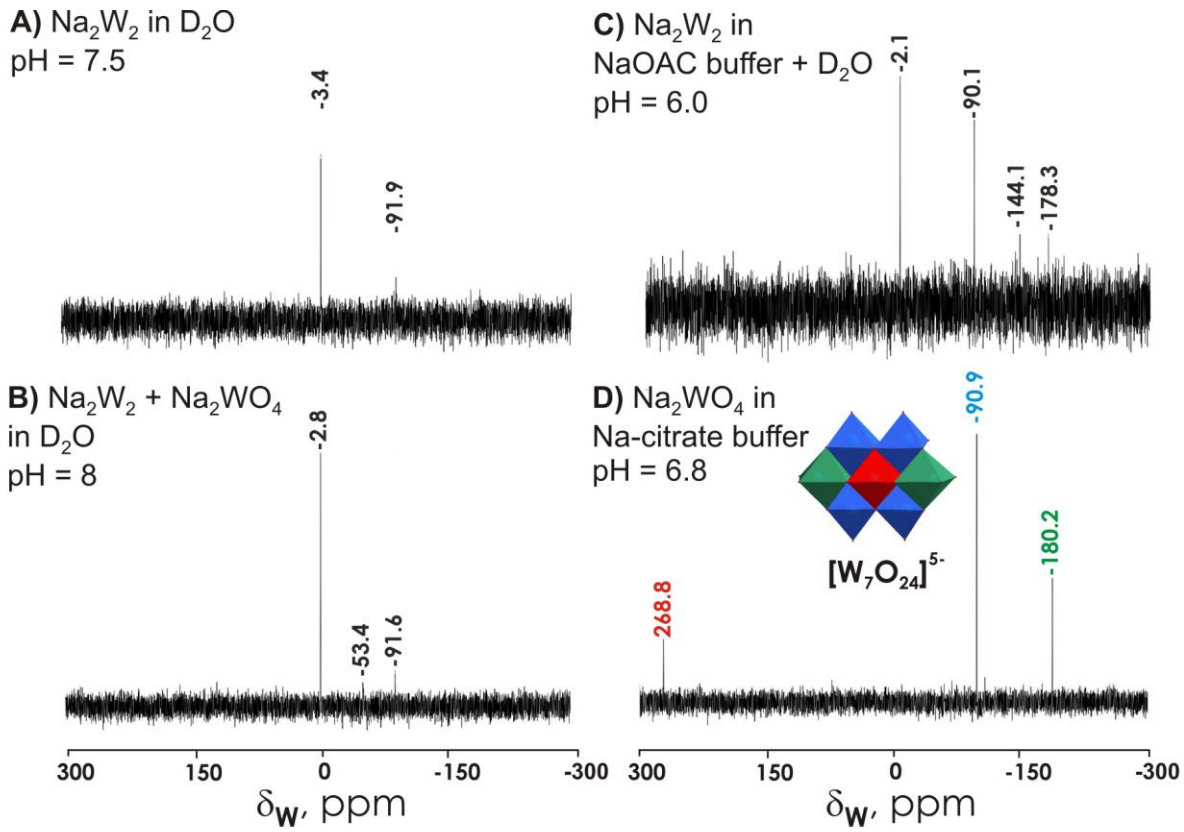

Figure S6. [183]W NMR spectra of $Na_2[W_2O_6(C_4O_3NH_{10})_2]\cdot 6H_2O$ (**Na_2W_2**) (150 mg in 3 mL of solvent, 0.058 M) solutions **A)** in D_2O with the final pH of 7.5; **B)** in D_2O with addition of 100 mg of $Na_2WO_4\cdot 2H_2O$; **C)** in NaOAc buffer (1.5 mL, pH 5.8) and D_2O (1.5 mL) with the final pH of 6.0; and [183]W NMR spectrum of $Na_2WO_4\cdot 2H_2O$ (150 mg in 3 mL of solvent, 0.058 M) solution **D)** in Na-citrate buffer pH 6.8 with 10% D_2O. The NMR signals in **D**) are assigned to W nuclei sharing the same chemical environment according to literature data.[10] The total recording time is ~ 60 h for each measurement; chemical shifts were measured relative to external 1 M Na_2WO_4. Color code: {WO_6}, blue, green, red.

Table S3. Experimental and calculated chemical shifts (ppm) for a selection of tungstates, including W_2. Shifts were calculated with the Gauge-Independent Atomic Orbital[11] method as implemented in Gaussian 16 rev. A.03[1] using the hybrid exchange–correlation functional PBE0 functional[2] and the all-electron TZP basis set by Jorge *et al*.,[3] on structures optimized at the PBE0/def2-tzvp level of theory.[11] All calculations us implicit solvation (water) via the polarizable continuum model.[12] Local minima were confirmed by the absence of negative vibrational modes.

Compound	Experimental shift (ppm)[13]	Shielding (Jorge-TZVP)	Calculated shift (ppm)
$[WO_4]^{2-}$	0	1885.6	0
WF_6	-1120	2581.1	-697
WCl_6	2181	-548.7	2433
$W(CO)_6$	-3505	5159.0	-3274
$[W_6O_{19}]^{2-}$	59	1799.6-1800.3	85
**W_2**	-3.4	1901.9	-17

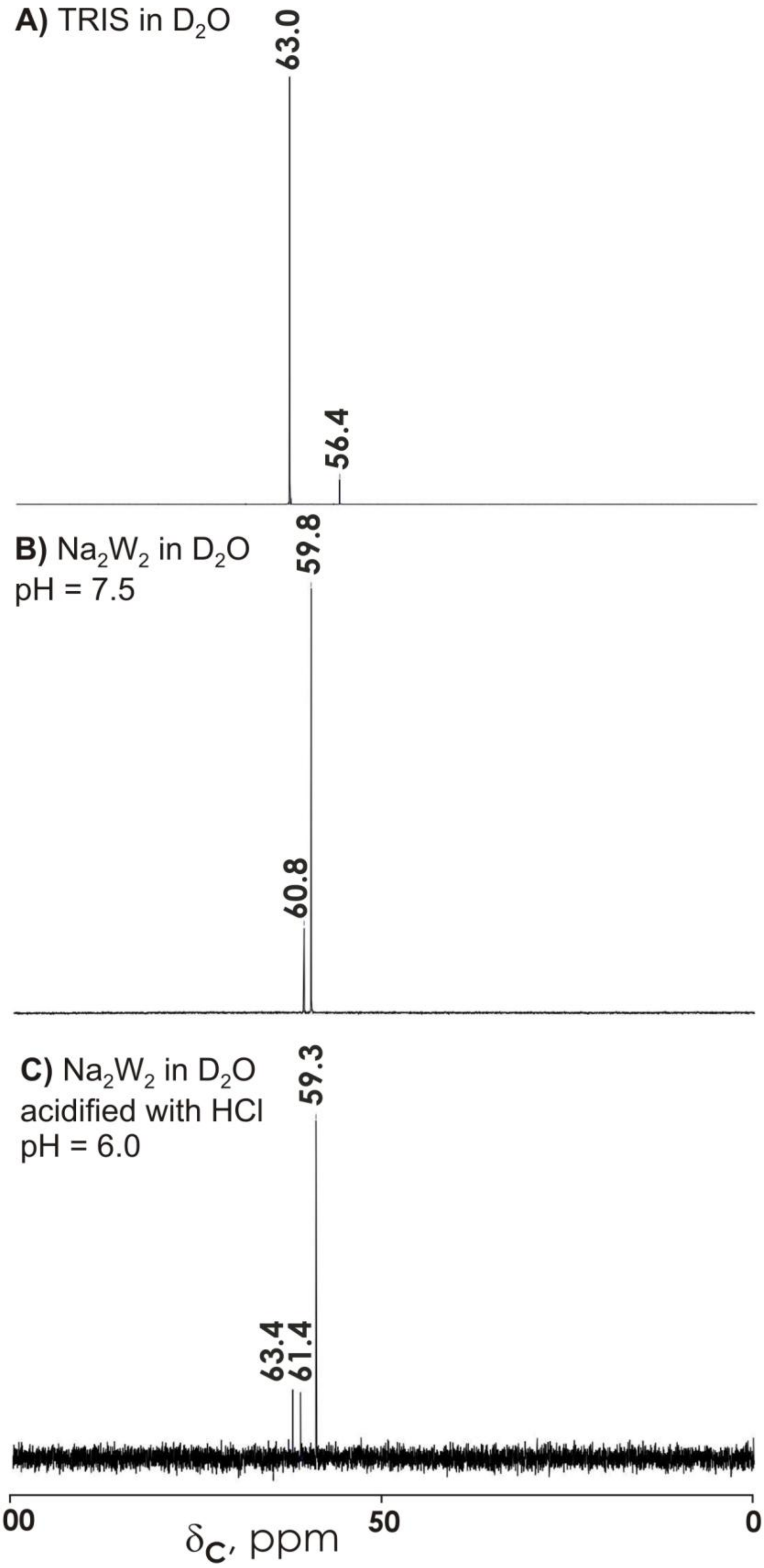

Figure S7. ^{13}C {^{1}H} NMR spectra of **A)** TRIS (30 mg in 0.6 mL of solvent) in D_2O; **B)** $Na_2[W_2O_6(C_4O_3NH_{10})_2]\cdot 6H_2O$ (**Na_2W_2**) (30 mg in 0.6 mL of solvent) in D_2O with the final pH of 7.5; **C)** $Na_2[W_2O_6(C_4O_3NH_{10})_2]\cdot 6H_2O$ (**Na_2W_2**) (30 mg in 0.6 mL of solvent) in D_2O acidified with HCl to pH of 6.0.

References

1 Gaussian 16, Revision A.03, Frisch, M. J.; Trucks, G. W.; Schlegel, H. B.; Scuseria, G. E.; Robb, M. A.; Cheeseman, J. R.; Scalmani, G.; Barone, V.; Petersson, G. A.; Nakatsuji, H.; Li, X.; Caricato, M.; Marenich, A. V.; Bloino, J.; Janesko, B. G.; Gomperts, R.; Mennucci, B.; Hratchian, H. P.; Ortiz, J. V.; Izmaylov, A. F.; Sonnenberg, J. L.; Williams-Young, D.; Ding, F.; Lipparini, F.; Egidi, F.; Goings, J.; Peng, B.; Petrone, A.; Henderson, T.; Ranasinghe, D.; Zakrzewski, V. G.; Gao, J.; Rega, N.; Zheng, G.; Liang, W.; Hada, M.; Ehara, M.; Toyota, K.; Fukuda, R.; Hasegawa, J.; Ishida, M.; Nakajima, T.; Honda, Y.; Kitao, O.; Nakai, H.; Vreven, T.; Throssell, K.; Montgomery, J. A., Jr.; Peralta, J. E.; Ogliaro, F.; Bearpark, M. J.; Heyd, J. J.; Brothers, E. N.; Kudin, K. N.; Staroverov, V. N.; Keith, T. A.; Kobayashi, R.; Normand, J.; Raghavachari, K.; Rendell, A. P.; Burant, J. C.; Iyengar, S. S.; Tomasi, J.; Cossi, M.; Millam, J. M.; Klene, M.; Adamo, C.; Cammi, R.; Ochterski, J. W.; Martin, R. L.; Morokuma, K.; Farkas, O.; Foresman, J. B.; Fox, D. J. Gaussian, Inc., Wallingford CT, 2016.

2 a) Perdew, J. P.; Burke, K.; Ernzerhof, M. Generalized gradient approximation made simple. *Phys. Rev. Lett.* **1996**, *77*, 3865-3868. DOI: 10.1103/PhysRevLett.78.1396. b) Adamo, C.; Barone, V. Toward reliable density functional methods without adjustable parameters: The PBE0 model. J. Chem. Phys. **1999**, *110*, 6158-6170. DOI: 10.1063/1.478522.

3 Jorge, F. E.; Canal Neto, A.; Camiletti, G. G.; Machado, S. F. Contracted Gaussian basis sets for Douglas-Kroll-Hess calculations: estimating scalar relativistic effects of some atomic and molecular properties. J. Chem. Phys. **2009**, *130*, 064108. DOI: 10.1063/1.3072360.

4 Schaefer, A.; Horn, H.; Ahlrichs, R. Fully optimized contracted Gaussian basis sets for atoms Li to Kr. *J. Chem. Phys.* **1992**, *97*, 2571-2577. DOI: 10.1063/1.4630967,

5 Bruker SAINT V8.32B Copyright © 2005-2015 Bruker AXS.

6 Sheldrick, G. M. **1996**. *SADABS.* University of Göttingen, Germany.

7 Sheldrick, G. M. A short history of SHELX. *Acta Cryst.* **2008**, *A64*, 112–122. DOI: 10.1107/S0108767307043930.

8 Dolomanov, O. V.; Bourhis, L. J.; Gildea, R. J.; Howard, J. A. K.; Puschmann, H. OLEX2: a complete structure solution, refinement and analysis program. *J. Appl. Cryst.* **2009**, *42*, 339–341. DOI: 10.1107/S0021889808042726.

9 Huebschle, C. B.; Sheldrick, G. M.; Dittrich, B. ShelXle: a Qt graphical user interface for SHELXL. *J. Appl. Cryst.* **2011**, *44*, 1281–1284. DOI: 10.1107/S0021889811043202.

10 Fan, L.; Cao, J.; Hu, C. What can electrospray mass spectrometry of paratungstates in an equilibrating mixture tell us? *RSC Adv.* **2015**, *5*, 83377–83382. DOI: 10.1039/C5RA18059G.

11 Ditchfield, R. Self-consistent perturbation theory of diamagnetism. 1. Gaug-invariant LCAO method for NMR chemical shifts. Mol. Phys. **1974**, 27, 789-807. DOI: 10.1080/00268977400100711.

12 Miertus; S., Scrocco, E.; Tomasi, J. Electrostatic interaction of a solute with a continuum. A direct utilization of ab initio molecular potentials for the prevision of solvent effects. Chem. Phys. **1981**, *55*, 117-129. DOI: 10.1016/0301-0104(81)85090-2.

13 Chen, Y.-G.; Gong, J.; Qu, L.-Y. Tungsten-183 nuclear magnetic resonance spectroscopy in the study of polyoxometalates, *Coord. Chem. Rev.* **2004**, *248*, 245–260. DOI: 10.1016/j.cct.2003.11.003.
